# Transfusion‐Associated Graft‐Versus‐Host Disease Risk and Transfusion Requirements After Cladribine in Multiple Sclerosis: Time to Revise Irradiation Policy?

**DOI:** 10.1111/ene.70698

**Published:** 2026-07-06

**Authors:** Klaus Schmierer, Frederick Chen, David Paling

**Affiliations:** ^1^ The Blizard Institute Queen Mary University of London, Faculty of Medicine & Dentistry London UK; ^2^ Clinical Board Medicine (Neuroscience) The Royal London Hospital, Barts Health NHS Trust London UK; ^3^ Clinical Haematology The Royal London Hospital, Barts Health NHS Trust London UK; ^4^ Sheffield Teaching Hospitals NHS Foundation Trust Royal Hallamshire Hospital Sheffield UK

**Keywords:** cladribine, disease modifying therapies, multiple sclerosis, transfusion‐associated graft‐versus‐host disease

## Abstract

**Background:**

Irradiation of cellular blood components prevents transfusion‐associated graft‐versus‐host disease (TA‐GVHD). UK and Spanish guidance recommend lifelong irradiation for all patients treated with purine analogues, including cladribine, irrespective of indication or dose.

**Objective:**

To assess whether this approach is justified for people with multiple sclerosis (pwMS) receiving cladribine.

**Methods:**

Narrative synthesis of immunological data, hemovigilance evidence, and national European transfusion guidelines.

**Results:**

Cladribine used in pwMS induces transient lymphopenia with modest, reversible T‐cell effects and preserved vaccine responsiveness. Since introduction of universal pre‐storage leucocyte depletion, hemovigilance data show no reported cases of TA‐GVHD in this setting. European guidance is heterogeneous, ranging from indefinite to time‐limited or unspecified durations.

**Conclusion:**

Current evidence does not clearly support the need for lifelong irradiation after cladribine treatment in pwMS. Time‐limited or individualized approaches merit consideration.

## 1 Introduction

Transfusion‐associated graft‐versus‐host disease (TA‐GVHD) is a rare but frequently fatal complication of transfusion of cellular blood components containing viable donor lymphocytes [1]. Donor T lymphocytes engraft within the recipient and mount an alloimmune response against recipient tissues, with mortality exceeding 90% in most reported series [1]. Historically, TA‐GVHD has been associated with profound T‐cell immunosuppression, directed donations from relatives, and populations with reduced HLA diversity, including Japan, where shared donor‐recipient HLA haplotypes may increase the likelihood of donor lymphocyte engraftment [2].

Irradiation of cellular blood components prevents donor T‐cell proliferation and has therefore become standard practice for selected high‐risk patient groups. However, transfusion medicine has evolved substantially over recent decades, particularly following the widespread introduction of universal pre‐storage leucocyte depletion, which markedly reduced the number of viable donor lymphocytes within transfused blood products [2, 3].

Current UK guidance nevertheless continues to recommend lifelong irradiation of cellular blood components for all patients treated with purine analogues, including cladribine, irrespective of indication or dose [3]. Whether this precaution remains justified for people with multiple sclerosis (pwMS) receiving contemporary low‐dose cladribine regimens warrants reconsideration.

Although blood transfusion is uncommon in routine neurological practice, the recommendation for lifelong irradiation affects all future transfusion episodes, emergency care pathways and perioperative management throughout a patient's lifetime.

## 2 Cladribine Immunology in Multiple Sclerosis

Cladribine is licenced for relapsing MS as an immune reconstitution therapy administered at a cumulative dose of 3.5 mg/kg over 2 years. Treatment is intermittent, comprising short oral treatment courses over approximately 8–10 days annually. This differs fundamentally from historical regimens used in hematological malignancies, where substantially higher cumulative doses, repeated treatment cycles, and combination chemotherapy were frequently employed. Importantly, many of the historical TA‐GVHD cases underpinning current recommendations occurred in patients with hematological malignancies receiving repeated courses of purine analogue therapy, often in combination with additional immunosuppressive agents.

In people with MS (pwMS), cladribine induces transient lymphopenia, predominantly affecting B cells, with comparatively modest and reversible reductions in CD4+ and CD8+ T cells [4, 5, 6, 7, 8, 9]. Lymphocyte nadirs typically occur several months after treatment initiation, followed by progressive immune reconstitution. Most patients recover to grade 0–1 lymphopenia within months after the final treatment course [9].

Importantly, cladribine does not produce continuous immunosuppression. Innate immune function is relatively preserved, and several studies have demonstrated preserved humoral responses and vaccine responsiveness, including responses to influenza, varicella zoster virus, and SARS‐CoV‐2 vaccination [6, 7, 8]. These observations support the concept that cladribine in MS produces selective and transient immune effects distinct from the profound and prolonged T‐cell depletion associated with many hematological regimens historically linked to TA‐GVHD (Table 1).

**TABLE 1:** Summary of evidence relevant to irradiation requirements after cladribine treatment in multiple sclerosis.

Cladribine effect	Observed TA‐GVHD risk	Implication for practice
Transient lymphopenia; modest T‐cell depletion; recovery within months	No reported cases post‐leucodepletion	Reconsider lifelong irradiation; consider time‐limited approach

## 3 Hemovigilance Evidence

Hemovigilance data following the introduction of universal prestorage leucocyte depletion demonstrate a marked reduction in TA‐GVHD incidence. UK surveillance data indicate that the overwhelming majority of reported cases occurred prior to leucodepletion, with only isolated cases subsequently reported and not clearly attributable to standard leuco‐depleted blood components [3].

Importantly, no cases of TA‐GVHD have been reported in association with cladribine use in pwMS despite extensive post‐marketing exposure. Although the rarity of TA‐GVHD limits definitive risk estimation, the residual risk in the contemporary leucodepletion era appears extremely low [2].

## 4 Comparison With Alemtuzumab

The precedent of alemtuzumab is instructive. Alemtuzumab produces substantially deeper and more prolonged T‐cell depletion than cladribine [9]. However, current guidance indicates that irradiation of blood components is not required when alemtuzumab is used according to MS protocols [3]. This discrepancy raises questions regarding the proportionality of current recommendations for cladribine.

## 5 European Guideline Comparison

National European recommendations for irradiation after purine analogue therapy are heterogeneous. UK guidance recommends lifelong irradiation for all patients treated with purine analogues, irrespective of indication or dose [3]. Spanish guidance similarly recommends indefinite irradiation [10]. In contrast, French and Italian guidance recommend time‐limited irradiation, typically for approximately 1 year after treatment [11, 12]. Dutch guidance suggests shorter durations (around 6 months) or relaxation of indications in the context of contemporary hemovigilance data [13]. German and Danish guidance recommend irradiation in association with purine analogue therapy but do not define a specific duration [14, 15]. Notably, no clear European consensus currently exists regarding the duration of irradiation after exposure to purine analogues (Table 2).

**TABLE 2:** European recommendations for irradiation after purine analogue therapy.

Country	Recommendation	Duration	Key references
UK	Yes	Indefinite	[3]
Spain	Yes	Indefinite	[10]
France	Yes	~1 year	[11]
Italy	Yes	≥ 1 year	[12]
The Netherlands	Yes	~6 months/relaxed	[13]
Germany	Yes	Not defined	[14]
Denmark	Yes	Not defined	[15]

## 6 Discussion

Taken together, these observations suggest that the continued recommendation for lifelong irradiation after cladribine treatment in pwMS may reflect historical precedent rather than a contemporary risk‐proportionate assessment.

The relatively modest and reversible T‐cell effects of cladribine in MS, combined with preserved vaccine responsiveness and the absence of reported TA‐GVHD cases in the leucodepletion era, contrast with the substantially more intensive purine analogue regimens historically used in hematological malignancy populations from which much of the original evidence derives.

The epidemiology of TA‐GVHD also differs geographically. Increased historical incidence in Japan has been attributed partly to reduced HLA diversity and increased donor‐recipient haplotype sharing [2]. These findings may not be directly generalizable to contemporary European transfusion practice. However, we agree that any modification of current recommendations should be interpreted in the context of regional epidemiology and HLA diversity.

The precedent of alemtuzumab remains particularly informative. Alemtuzumab induces substantially deeper and more prolonged T‐cell depletion than cladribine [9], yet current UK guidance does not require irradiation of blood components when alemtuzumab is used according to MS protocols [3]. From a risk‐proportionate perspective, this discrepancy is difficult to reconcile.

International guidance further underscores the absence of consensus. Whereas UK and Spanish recommendations favour indefinite irradiation, several European countries now advocate time‐limited approaches or provide no defined duration [10, 11, 12, 13, 14, 15].

## 7 Conclusion

Current evidence does not clearly support the need for lifelong irradiation after cladribine treatment in pwMS and supports reconsideration of this requirement. At minimum, time‐limited approaches may be more consistent with contemporary immunological understanding and hemovigilance data.

## Author Contributions

**Klaus Schmierer:** conceptualization, investigation, methodology, writing – original draft, supervision. **Frederick Chen:** writing – review and editing, methodology, investigation. **David Paling:** investigation, methodology, writing – review and editing, conceptualization.

## Conflicts of Interest

K.S. has received speaker honoraria from Neuraxpharm, Novartis, Roche, Sanofi, the healthcare business of Merck KGaA, Darmstadt, Germany, and EMD Serono Research & Development Institute, Inc., Billerica, MA, an affiliate of Merck KGaA, Darmstadt, Germany; research grants, through Queen Mary University of London, from Biogen, JUVISE, the healthcare business of Merck KGaA, Darmstadt, Germany, Novartis, Roche and Sandoz; he has received service support, through Queen Mary University of London and Barts Health NHS Trust, from Neuraxpharm. F.C. has no conflicts of interest. D.P. is the principal investigator for commercial trials funded by Novartis, Merck, Janssen Pharmaceuticals and Roche; chief investigator for commercial trials funded by Novartis; has an investigator grant from Sanofi Genzyme; and has received advisory board/consultancy and speaker's fees from Biogen, Celgene, Janssen, MedDay, Merck, Novartis, Roche and Sandoz.

## Data Availability

Data sharing not applicable to this article as no datasets were generated or analysed during the current study.
